# No Time to Waste: Real-World Repurposing of Generic Drugs as a Multifaceted Strategy Against COVID-19

**DOI:** 10.2196/19583

**Published:** 2020-09-30

**Authors:** Moshe Rogosnitzky, Esther Berkowitz, Alejandro R Jadad

**Affiliations:** 1 Medinsight Research Institute Rehovot Israel; 2 Program in Impactful Giving Dalla Lana School of Public Health University of Toronto Toronto, ON Canada

**Keywords:** COVID-19, drug repurposing, fibrates, histamine type-2 receptor antagonists, cimetidine, famotidine, fenofibrate, bezafibrate, dipyridamole, sildenafil

## Abstract

Real-world drug repurposing—the immediate “off-label” prescribing of drugs to address urgent clinical needs—is an indispensable strategy gaining rapid traction in the current COVID-19 crisis. Although off-label prescribing (ie, for a nonapproved indication) is legal in most countries, it tends to shift the burden of liability and cost to physicians and patients, respectively. Nevertheless, in urgent public health crises, it is often the only realistic source of a meaningful potential solution. To be considered for real-world repurposing, drug candidates should ideally have a track record of safety, affordability, and wide accessibility. Although thousands of such drugs are already available, the absence of a central repository of off-label uses presents a barrier to the immediate identification and selection of the safest, potentially useful interventions. Using the current COVID-19 pandemic as an example, we provide a glimpse at the extensive literature that supports the rationale behind six generic drugs, in four classes, all of which are affordable, supported by decades of safety data, and pleiotropically target the underlying pathophysiology that makes COVID-19 so dangerous. Having previously fast-tracked this paper to publication in summary form, we now expand on why cimetidine/famotidine (histamine type-2 receptor antagonists), dipyridamole (antiplatelet agent), fenofibrate/bezafibrate (cholesterol/triglyceride-lowering agents), and sildenafil (phosphodiesterase-5 inhibitor) are worth considering for patients with COVID-19 based on their antiviral, anti-inflammatory, renoprotective, cardioprotective, and anticoagulation properties. These examples also reveal the unlimited opportunity to future-proof public health by proactively mining, synthesizing, and cataloging the off-label treatment opportunities of thousands of safe, well-established, and affordable generic drugs.

## Introduction

Since the first report of a viral pneumonia of unknown cause in Wuhan, China, in December 2019, followed by the identification of the virus SARS-CoV-2 and the designation of the disease it causes as COVID-19, we have witnessed the rapid development of a pandemic that has become a global public health crisis. Although reported mortality rates are between <1% and 27% depending on factors including age, gender, health status, and geographic location, this is likely an underestimation due to underreporting and limited serological testing [1]. With no approved preventive or therapeutic treatments available, the scale and human impact of the COVID-19 outbreak is daunting.

In this paper, we present candidates for a multifaceted approach to the management of COVID-19, based on repurposing tried and tested, affordable, widely available drugs with proven long-term safety, and mechanisms of action that address the underlying pathophysiology of the disease. Having recently published a short summary of our thesis [2], the current paper expands on why cimetidine or famotidine (histamine type-2 receptor antagonists), dipyridamole (antiplatelet agent), fenofibrate or bezafibrate (cholesterol/triglycerides-lowering agents), and sildenafil (phosphodiesterase-5 inhibitor) are worth considering for patients with COVID-19. The goal is to enable the rapid introduction of potentially beneficial, low-risk interventions. We also emphasize the urgency of redoubling efforts to mine, synthesize, and catalog the considerable existing body of evidence for promising treatments, in order to future-proof public health, based on robust science.

## Pathophysiology of COVID-19

COVID-19 is characterized by prominent early respiratory signs and symptoms, including fever, cough, fatigue, and shortness of breath, that may deteriorate to acute respiratory distress syndrome (ARDS), coagulopathy, multiorgan failure, and other life-threatening sequelae (Table 1) [3,4]. On lung imaging, consolidation, ground glass opacity, and pulmonary infiltration are evident [3].

**Table 1:** Common clinical findings, complications, and laboratory abnormalities in patients with laboratory-confirmed COVID-19. CRP: C-reactive protein.

System and clinical finding		Prevalence (%)
**Respiratory [3,4]**		
	Fever	79-98
	Cough	58-79
	Respiratory failure	54
	Acute respiratory distress syndrome	30
	Sputum production	12-28
**Cardiovascular [3]**		
	Cardiac failure	23
	Septic shock	20
**Multisystem [3,4]**		
	Sepsis	59
	Fatigue	20-44
	Coagulopathy	19
**Laboratory abnormalities [3,4]**		
	Lactate dehydrogenase >245 U/L	67-73
	Procalcitonin <0.1 ng/mL	70
	D-dimer >1 μg/mL	65-70
	Lymphopenia	40-63
	Aspartate aminotransferase > 40 U/L	37
	Leucopenia	22-25
	Raised CRP	—^a^

^a^Not available.

SARS-CoV-2 has also been isolated from feces, urine, blood, and ophthalmic secretions [5]; COVID-19 affects extrapulmonary organs and systems in ways that contribute significantly to overall morbidity and mortality. In a retrospective cohort study of 191 hospitalized patients with COVID-19 in Wuhan, sepsis was reported in 112 (59%) patients admitted to the hospital, while respiratory failure (54%), ARDS (31%), heart failure (23%), and septic shock (20%) were reported in ≥20% of patients, significantly more frequently among those who died than in survivors (all *P*<.001) [3]. Similarly, coagulopathy (19%) and acute cardiac (17%) and renal (15%) injury were widely observed, with a significantly higher incidence in nonsurvivors [3]. The fact that underlying cardiovascular, pulmonary, and renal disease have been associated with significantly increased mortality in COVID-19 patients and that abnormalities of various plasma inflammatory biomarkers (eg, lymphocyte count, C-reactive protein (CRP), procalcitonin, D-dimer, and aspartate aminotransferase) appear to be widespread [3,4,6,7] highlights the multisystem nature of the disease and suggests that immune-mediated cytokine signaling and development of cytokine storm play a key role in driving disease progression [7].

Laboratory findings support the diverse effects of SARS-CoV-2, demonstrating, among other derangements, that leukopenia, lymphopenia, and thrombocytopenia, as well as elevated lactate dehydrogenase, CRP, and D-dimer, are significantly associated with a more severe course of disease [3,4,6,8]. Viral load also correlates strongly with disease severity (lung injury in particular) [9], while virus-induced endothelial dysfunction contributes to acute cardiac events that are a recognized complication of COVID-19 [10]. Although little attention has focused specifically on disturbed coagulation, evidence suggests that COVID-19 leads to profoundly altered coagulation function, with raised D-dimer, fibrinogen, and fibrin/fibrinogen degradation products [11,12].

## Potential Therapies Within the Current Pharmacopoeia

Widespread attention has been given to repurposing antimalarial chloroquine/hydroxychloroquine, the antibiotic azithromycin and, most recently, the antiparasitic agent ivermectin, for targeting COVID-19. All three drugs possess both anti-infective and immune-modulating properties [13-19]. While clinical evidence supporting their use individually or in combination in patients with COVID-19 have so far been inconclusive [13-19], partly due to methodological limitations, or are yet unavailable [20-22], this multitargeted approach of utilizing the safest drugs with pleiotropic effects is essential to reduce morbidity and mortality arising from COVID-19 infection. Indeed, a large number of approved drugs have mechanisms of action that could be harnessed to address the pathophysiology of COVID-19. Ideal choices would be safe and widely available generic drugs that are affordable in any setting, and especially for under-resourced populations. Below we summarize the safety profiles and rationale for repurposing several generic drugs that have demonstrated antiviral, anti-inflammatory, and/or cardio-, lung- or renal-protective effects. Some also lower elevated fibrinogen and D-dimer, which are associated with hypercoagulability and may contribute to the multiorgan failure seen in patients with COVID-19. Table 2 summarizes the physiological effects of these agents as they relate to potential benefits in patients with COVID-19.

**Table 2:** Approved indications and recognized physiological effects of drugs that could be considered for repurposing in patients with COVID-19.

Drug	Current indications	Proposed dose	Demonstrated effects^a^							Notes
			A	B	C	D	E	F	G	
Cimetidine or famotidine [23-46]	Symptomatic management of GERD^b^	Cimetidine 200 mg four times daily Famotidine 20-40 mg twice daily			✓		✓			Establish baseline prolactin levels and monitor periodically May increase serum concentrations of other drugs Reduces dipyridamole absorption Relevant trials: NCT04504240 and NCT04370262
Dipyridamole [47-79]	Antithrombotic following cardiac valve replacement	75 mg thrice daily OR 50-100 mg once weekly	✓	✓	✓	✓	✓	✓		May cause headaches during the first week of use Taking with foods or antacids halves absorption Relevant trials: NCT04391179, NCT04424901, and NCT04410328
Fenofibrate or bezafibrate [80-97]	Dyslipidemia	Fenofibrate ≤200 mg/day Bezafibrate 400 mg daily		✓	✓	✓	✓	✓		Significant reduction in D-dimer and fibrinogen usually seen in days Relevant trial: NCT04517396
Sildenafil citrate [98-112]	Erectile dysfunction	25 mg twice daily, on an empty stomach		✓			✓	✓	✓	Avoid grapefruit juice (increases sildenafil levels) Cimetidine/famotidine increases sildenafil concentration. If combined, consider lower sildenafil dose —even 12.5 mg twice daily Relevant trials: NCT04304313 and NCT04489446

^a^Demonstrated effects are: A - preliminary efficacy in COVID-19 patients; B - anti-inflammatory effect; C - antiviral effect; D - anticoagulant effect; E - cardioprotective effect; F - renoprotective effect; G - lung protective effect.

^b^GERD: gastroesophageal reflux disease.

### Cimetidine and Famotidine

The histamine type-2 receptor antagonists (H_2_RAs) cimetidine and famotidine were approved by the US Food and Drug Administration (FDA) in 1977 and 1986, respectively, and both have been widely used, for decades, for prevention and symptomatic management of gastroesophageal reflux disease (GERD) [23]. Ranitidine, another commonly used H_2_RA, will soon be largely unavailable in the United States following an FDA recall based on high levels of a contaminant. Cimetidine is approved at daily doses of 200-400 mg for heartburn relief, and up to 1600 mg for the short-term treatment of erosive GERD, while famotidine is approved at a dose of 10-20 mg twice daily (bid) for GERD, and 20-40 mg bid for erosive esophagitis [23]. Both drugs are available over the counter in the United States and much of the world, and are generally well tolerated, with most adverse events reported in <1% of patients. Drug-drug interactions that may delay metabolism of other agents due to interaction with the cytochrome P450 system, limited antiandrogen effects, and stimulation of prolactin are recognized in particular with cimetidine. Prolactin levels should therefore be established at baseline and monitored periodically.

### Antiviral and Immunomodulatory Activity

Beyond their role as gastric acid reducers, H_2_RAs have powerful modulatory effects on innate and adaptive immunity by interfering with the effects of histamine on a range of leukocytes. As such, they reverse histamine-mediated immunosuppression by stimulating the effector functions of a wide range of T and B cells [24]. The resulting antiviral effects have been demonstrated in small studies in patients with herpes simplex virus (HSV) [25] and herpes zoster infection [26], and with human papillomavirus–related disorders [27]. In preclinical studies, cimetidine boosted immune cellular response when used as an adjuvant to viral vaccines for hepatitis B virus [28-31] and suppressed HIV replication in vitro [32]. Furthermore, intravenous ranitidine significantly increased the antibody response to vaccination in patients receiving tetanus toxoid before major abdominal surgery [33] and in patients with B-cell chronic lymphocytic leukemia receiving tetanus toxoid–conjugated *Haemophilus influenzae* type b vaccine [34,35]. Beyond antiviral effects, H_2_RAs have shown immunomodulatory effects in a range of cancers and allergic diseases, bone resorption, and during recovery from burn injury [24].

### Cardiovascular Protective Effects

H_2_RAs also demonstrate a number of cardioprotective effects. A meta-analysis of 10 randomized controlled trials in patients with congestive heart failure, most of whom used famotidine, showed that orally administered H_2_RAs were associated with significant negative inotropic and chronotropic effects (reduction in heart rate vs placebo; *P*=.02), and also significantly decreased blood pressure and increased cardiac efficiency, presumably reducing myocardial oxygen requirement [36]. In another study, in critically ill patients, a single intravenous infusion of cimetidine, 200 mg, reduced systolic, diastolic, and mean arterial blood pressure, and raised heart rate [37]. High-dose intravenous cimetidine (200 mg four times daily [qid]) administered after elective cardiac bypass surgery was also shown to reduce levels of proinflammatory mediators (neutrophil elastase, interleukin-8, CRP) with no adverse effects, suggesting the potential to improve cardiac outcomes under certain physiological conditions [38].

Furthermore, H_2_RAs also strongly inhibit platelet aggregation in vitro [39,40] and ranitidine, in combination with hydrocortisone, has been shown to reduce complications after arterial thrombolysis in pediatric patients who developed arterial obstruction after cardiac catheterization [41]. These agents may therefore exert stabilizing effects on coagulation in patients with disturbed clotting function, the caveat being the potential for thrombocytopenia and/or hemolytic anemia [42-46].

### Dipyridamole

The antiplatelet agent and phosphodiesterase inhibitor dipyridamole was first approved in 1961 and is indicated in the United States at doses of 75-100 mg qid with warfarin to decrease thrombotic risk following cardiac valve replacement [47]. It is also sold in the United States as a combined product, Aggrenox (aspirin 25 mg/extended-release dipyridamole 200 mg), which was approved in 1999 and is taken twice daily to reduce stroke risk [48]. Outside the United States, including in Europe, dipyridamole is available as a single agent; in Russia it is also approved as an antiviral agent [49]. Within the 200-400 mg daily dose range, dipyridamole is considered safe based on decades of clinical experience: adverse events are usually limited and transient, the most common being dizziness, gastrointestinal disturbance, headache, and skin rash [50]. The pleiotropic effects of dipyridamole derive from increased intracellular levels of cyclic adenosine monophosphate (cAMP) and cyclic guanine monophosphate (cGMP), which lead to anti-inflammatory, antioxidant, anticoagulation, and vasodilatory effects [51].

### Antiviral Effects

Dipyridamole possesses very broad spectrum antiviral activity as shown in numerous preclinical studies that demonstrated efficacy, alone or as a potentiator of other agents, against HSV, HIV, varicella zoster, cytomegalovirus, and mengovirus, as well as a range of viruses from the picornavirus, togavirus, orthomyxovirus, paramyxovirus, and pox virus families [52-56]. Induction of interferon responses have been identified as an important contributory factor to its antiviral effects [57]. A number of clinical studies have confirmed the antiviral effects of dipyridamole which, at a dose of 8-100 mg weekly, significantly reduced the risk of acute respiratory diseases, including influenza, when administered prophylactically to at-risk individuals [57-59].

### Effects in Patients With COVID-19

A recent study in patients with COVID-19 in China illustrates that dipyridamole suppressed SARS-CoV-2 replication in vitro, induced potent antiviral immunity, and improved survival in a mouse model of pneumonia [60]. In a clinical study of 12 COVID-19 patients that was conducted alongside these preclinical investigations and reported within the same publication, dipyridamole increased lymphocyte and platelet counts, decreased D-dimer levels, and markedly improved clinical outcomes when dosed at 50 mg three times daily for 1 week. In this small but very promising study, three of the six patients with severe disease were discharged, and four (33%) mild cases achieved clinical remission. Data from an ongoing multicenter study examining dipyridamole in 460 patients with COVID-19 in China (ChiCTR2000030055) will add to our understanding [61].

### Anti-Inflammatory, Antioxidant, and Endothelial Protective Effects

A large number of preclinical studies have demonstrated that dipyridamole limits oxidative stress in platelets and endothelial cells, inhibits release of proinflammatory cytokines, and reduces inflammatory responses, independent of its antiplatelet activity [51,62]. This has implications for a wide range of pathologies beyond thrombosis prevention, including reduced brain endothelial injury after inflammatory and metabolic insult [63]. The combination of dipyridamole with prednisolone has been shown to lead to significant reductions in interferon-γ, interleukin-6, and CRP in subjects with periodontitis [64], and widening of the therapeutic window of glucocorticoid activity [65]. This is due to the ability of dipyridamole to selectively potentiate the effects of prednisolone and other glucocorticoids. Dipyridamole was also shown to increase extracellular levels of the immune-dampening nucleoside, adenosine, and decrease CD4+ T cell activation by 11.1% (*P*=.006) in patients with chronic HIV infection receiving antiviral therapy [66].

### Antihypercoagulation Effects

Hypercoagulability is a potentially life-threatening complication of certain clinical conditions and a serious risk during mechanical circulatory support. Besides its well-established antiplatelet effects, dipyridamole has shown efficacy as one component of a near-universal anticoagulant when administered in combination with citrate, theophylline, and adenosine (as CTAD [citrate-theophylline-adenosine-dipyridamole]) in veterinary practice [67,68] and in human subjects [69]. When combined with heparin or aspirin in small numbers of pediatric patients on circulatory support [70] or with disseminated intravascular coagulation [71], dipyridamole has led to clinical recovery in the majority of subjects.

### Cardioprotective Effects

Adenosine serves as an endogenous cardioprotective agent. Disturbances of adenosine in the diseased myocardium include raised plasma levels and decreased gene expression of certain receptors in patients with chronic heart failure [72,73] as well as impaired vasodilation in patients with hyperhomocysteinemia [74]. By increasing adenosine levels in vivo using dipyridamole ≤300 mg daily, it was possible to improve numerous functional measures of disease severity in cases of chronic heart failure [72,73] and restore adenosine-induced vasodilation in hyperhomocysteinemic patients [74], highlighting the potential to augment endogenous cardioprotective mechanisms. The clinical effects of dipyridamole in mild-to-moderate chronic heart failure were also revealed in a trial that randomized 28 patients to their usual treatment with or without the addition of dipyridamole, 75 or 300 mg/day, for 1 year [75]*.* Cardiac ejection fraction, left ventricular systolic diameter, maximal oxygen consumption, and plasma B-type natriuretic peptide level were all significantly improved versus baseline and control in dipyridamole-treated patients, in a broadly dose-dependent manner, indicating a role for supplementary dipyridamole in improving the pathophysiology of chronic heart failure.

### Renoprotective Effects

In patients with kidney disease, dipyridamole reduces proteinuria and improves renal function by inhibiting platelet activation and enhancing nitric oxide (NO)–induced vasodilation. A prospective study of >28,000 patients with advanced chronic kidney disease (CKD) in Taiwan found that dipyridamole significantly reduced the risk of progression to long-term dialysis and predialysis death (hazard ratios 0.96 and 0.91, respectively; both *P*<.05 versus nonuse) [76]. In another large Taiwanese study in patients with advanced CKD, dipyridamole decreased the risk of progression to end-stage renal disease by approximately 15% and reduced all-cause mortality by 23.5% (*P*=.001) [77]. There is also evidence that the vascular renoprotective effects may benefit patients with immunoglobulin A nephropathy (when given with warfarin) [78] and protect against preeclampsia [79].

### Fenofibrate and Bezafibrate

The cholesterol-lowering agents fenofibrate and bezafibrate are indicated for the treatment of dyslipidemias. Fenofibrate is a peroxisome proliferator–activated receptor-α agonist that was approved in the United States in 1993 and is used for the treatment of primary hypertriglyceridemia, mixed dyslipidemia, and severe hypertriglyceridemia (up to 160 mg once daily) [80]. Abnormal liver tests, elevated liver enzymes and creatine phosphokinase, and rhinitis are the most frequent adverse events. Rare instances of myositis or rhabdomyolysis have been reported, and potentiation of coumarin anticoagulants can cause bleeding, so a reduced anticoagulant dose is advised [80]. Bezafibrate is currently not approved for use in the United States but is commonly used in Europe.

Numerous preclinical studies support a role for fenofibrate in attenuating vascular endothelial dysfunction, oxidative stress, and inflammation across a range of organs and tissues, with clinical evidence of cardioprotection and some antiviral effects [81].

### Antiviral Effects

A meta-analysis of eight observational studies of fibrates, with or without statins, in patients with hepatitis C virus infection, revealed a significant reduction in viral load, with bezafibrate demonstrating the greatest antiviral efficacy among the medications tested. The antiviral potency of bezafibrate was confirmed in both Asian and European study populations. Interestingly, the significant clinical effect was found despite the failure of in vitro studies to demonstrate a significant effect [82].

### Anti-Inflammatory Effects

The anti-inflammatory properties of fibrates that underpin some of the macrovascular benefits also translate into improved clinical outcomes in patients with microvascular disease. In in vitro studies, fenofibrate demonstrated potentially protective effects on the renal and retinal microvasculature by suppressing inflammation and apoptosis in human glomerular microvascular cells and reducing retinal microvascular inflammation [83,84], while bezafibrate decreased the number of circulating proinflammatory monocytes in patients with type 2 diabetes [85]. A beneficial modulatory role for fenofibrate in renal fibrosis and inflammation has also been proposed, with further studies required to elucidate the mechanisms involved [81].

### Cardioprotective Effects

Preclinical studies provide evidence that fenofibrate can protect against cardiac ischemia-reperfusion injury and subsequent arrhythmias and heart failure, autoimmune myocarditis, and hypertension [81,86,87]. In the clinical setting, a meta-analysis of studies in which fibrates were used for primary prevention of atherosclerotic cardiovascular disease reported a significant 16% decrease in the combined outcome of cardiovascular death, nonfatal myocardial infarction, and nonfatal stroke [88] while a 12% reduction in these outcomes was reported when fibrates were used in secondary prevention [89].

### Anticoagulation Effects

Robust data indicate that fibrates lower plasma fibrinogen levels to a significant degree, independent of their lipid-lowering effects [90-95]. In a meta-analysis of 22 trials, representing >2700 participants, fibrates (fenofibrate and bezafibrate) demonstrated a significantly greater effect than statins in lowering plasma fibrinogen concentrations (weighted mean difference –40.7mg/dL, *P*<.001) [93]. Data from smaller studies focusing on the antihyperlipidemic effects of fibrates (fenofibrate 200 mg daily; bezafibrate 400 mg daily) show concurrent, significant reductions in plasma fibrinogen levels [90-92,94]. Of note is a study that reported an increase in fibrinogen levels among patients taking statins [94], which could be a concern in elderly patients with COVID-19, many of whom are likely to be taking statins.

In patients with metabolic syndrome, which represents a hypercoagulable state accompanied by inflammation and endothelial dysfunction, fenofibrate reduced concentrations of thrombin-activatable fibrinolysis inhibitor, improved endothelial function [96], and significantly reduced fibrinogen and D-dimer concentrations [97], suggesting potential anticoagulant and cardiovascular protective effects. This potential was borne out in a short-term randomized controlled trial of patients with acute ST-elevation myocardial infarction, in whom bezafibrate lowered fibrinogen concentration more effectively than conventional therapy (*P*<.001), with significantly greater reductions in the incidence of angina (56% vs 4%, *P*<.001) and left ventricular failure (24% vs 4%, *P*=.049) [95].

### Sildenafil Citrate

The phosphodiesterase-5 inhibitor (PDE5 inhibitor) sildenafil citrate is a vasodilator that was approved by the FDA in 1998 for the treatment of erectile dysfunction, at a dose of 25-100 mg once daily [98]. More recently, an indication for pulmonary arterial hypertension (PAH) was added in 2005, with an oral dosing of 5 or 20 mg thrice daily, or 2.5 mg or 10 mg as intravenous bolus [99]. In erectile dysfunction, orally administered sildenafil has an onset of action within 30 minutes, maximum effect at 1 hour, and duration of effect of 4-6 hours; in PAH, the pharmacodynamics are similar although peak effect occurs 1-2 hours after dosing, and blood pressure levels return to baseline levels within 8 hours [98,99]. The most common dose-dependent adverse reactions (≥5%) include headache, flushing, dyspepsia, visual disturbance, and nasal congestion. CYP3A4 inhibitors are known to potentiate sildenafil, while sildenafil potentiates the hypotensive effects of nitrates and alpha-blockers.

Sildenafil inhibits breakdown of cGMP through competitive binding at the phosphodiesterase binding site. It therefore influences platelet activation, proliferation of T cells, and production of proinflammatory cytokines, leading to a broad range of anti-inflammatory, antioxidant, vasodilatory, and other actions in many body systems [100]. An ongoing phase 3 trial of sildenafil, 100 mg daily for 14 days, in patients with COVID-19 (NCT04304313) will help clarify its potential benefits in this disease [101].

### Immunomodulatory Effects

In vitro human studies indicate that sildenafil potentiates the ability of regulatory T cells to downregulate T effector cell proliferation, while clinical findings include reduced lymphocyte count and induction of malignant cell apoptosis in a patient with B-cell chronic lymphocytic leukemia and in patients with Waldenstrom’s macroglobulinemia [100]. It was hypothesized that these effects were mediated by synthesis and release of cytokines. Sustained increase in NO production, and decreased vascular inflammatory markers, have also been reported in patients with type 2 diabetes receiving sildenafil [102,103].

### Cardiovascular Protective Effects

One-time and long-term administration of PDE5 inhibitors in patients at high cardiovascular risk can improve endothelial function, reduce inflammatory mediators, and increase endothelial regenerative capacity, which may be sustained for several months following treatment discontinuation, with potential applications in a range of cardiovascular disorders [104,105]. Cardioprotective effects include improved symptoms and cardiac contractility in patients with systolic heart failure, reduced myocardial infarct size, reduced blood pressure, and limitation of ischemia-driven ventricular arrhythmias, with reduction in cardiovascular events and mortality in high-risk patients [106-108]. In a British study that followed nearly 6000 men with type 2 diabetes over 7.5 years, the use of PDE5 inhibitors was associated with lower mortality risk overall (adjusted hazard ratio 0.54, *P*=.002) and in those with a history of acute myocardial infarction (heart rate=0.60, *P*=.001) [108]. These effects are believed to result from improved pulmonary circulation, as well as direct action on the myocardium, independent of the vasculature [106].

### Lung-Protective Effects

Studies demonstrating sildenafil’s efficacy and tolerability in PAH continue to accrue, and a 2019 Cochrane systematic review and meta-analysis comprising 36 studies of nearly 3000 patients concluded that those with PAH who received PDE5 inhibitors were 22% less likely to die in the short-term than those receiving placebo [109]. Additionally, a network meta-analysis reported moderate-level evidence that sildenafil may reduce mortality in idiopathic pulmonary fibrosis, an interstitial lung disease with high mortality [110]. A single case report of a 55-year-old physician with an atypical respiratory infection and apparently normal pulmonary arterial blood pressure who experienced marked symptomatic and functional improvement within 24 hours of starting tadalafil highlights the potential benefits of PDE5 inhibitors in this indication [111].

### Renoprotective Effects

Preliminary evidence suggests that the clinical efficacy of PDE5 inhibitors in CKD extends beyond antihypertensive effects to active renoprotection. In preclinical studies, PDE5 inhibitors suppressed mesangial cell proliferation and extracellular matrix expansion, reduced renal cell apoptosis, and decreased oxidative stress and inflammation [107]. A post hoc examination of this class of medications in a randomized controlled trial also revealed improved kidney function and functional capacity, and a trend toward reduced mortality, in patients with PAH who received sildenafil treatment [112]. Few, if any, clinical studies of PDE5 inhibitors in patients with acute kidney disease have been published. Ongoing clinical trials (eg, NCT04304313) will shed further light on this and may reveal information that could be applied in the treatment of patients with COVID-19 [101].

## Conclusions

Under the extraordinary COVID-19 pandemic conditions that have brought the world to the brink of an irreversible crisis, time is of the essence for the success of life-saving efforts. Until a vaccine is developed to treat this disease, the urgency of finding safe and effective treatments cannot be overstated. To ensure that patients with COVID-19 have rapid access to safe treatments, and to ensure the responsible use of available resources, it would be wise to mine the existing pharmacopeia for safe generic drugs that address the pathophysiologies underlying COVID-19. Moreover, beyond the current emergency there remains the likelihood of future re-emergence of another coronavirus or similar virus. The efforts we make now to facilitate access to information on the off-label applications of well-understood drugs, regardless of the manner in which the information has been discovered, are an investment in our future health that also addresses current needs. While clinical trials to assess efficacy will be important in due course, judicious use of one or more of these approved drugs, with caution toward potential interactions with concomitant medications, represents a rational and ethical approach that may prove effective in the short term. There is no time to waste and little to lose.

## Epilogue

Since the initial submission of this article as a preprint in April 2020, new developments and evidence have emerged that further support the therapeutic potential of the drugs proposed in this paper for use in the treatment of COVID-19. The new developments and evidence are summarized below and are current as of August 31, 2020.

### Dipyridamole

Research aimed at assessing the therapeutic potential of dipyridamole continues. One ongoing phase 3 clinical trial (ClinicalTrials.gov ID NCT04410328) randomized patients (n=132) to receive dipyridamole ER 200 mg and aspirin 25 mg orally/enterally plus standard care or standard care alone [113]. Researchers are also evaluating dipyridamole in two other ongoing clinical trials with a focus on determining the extent to which the drug can reduce excessive coagulation [114] and treat respiratory tract infection and circulatory dysfunction caused by SARS-CoV-2 [115] in hospitalized COVID-19 patients.

### Famotidine

The therapeutic potential of famotidine (combined with cetirizine) in COVID-19 treatment was recently boosted by an American cohort study evaluating the efficacy of dual-histamine blockade in patients with COVID-19. In the study, hospitalized COVID-19 patients with severe-to-critical symptoms were treated with cetirizine 10 mg and famotidine 20 mg bid in addition to standard care. This combination reduced symptom progression when compared to published reports of COVID-19 patients [116]. The safety and efficacy of famotidine in COVID-19 is further supported by a case series of 10 US patients with COVID-19 who self-administered high-dose oral famotidine (80 mg thrice daily was the most frequent regimen used) for 11 days. All patients reported marked improvements in COVID-19–related symptoms, suggesting that high-dose oral famotidine is well tolerated and associated with improved patient-reported outcomes in nonhospitalized patients with COVID-19 [117].

Another case series of 14 COVID-19 hospitalized patients from Beloit Memorial Hospital, United States, reported improvement in supplemental oxygen requirements, ground-glass computed tomography findings, and serum levels of lactate dehydrogenase, ferritin, CRP, D-dimer, and lymphocytes in patients who received famotidine 80 mg qid plus celecoxib (as adjuvant therapy) [118]. This treatment combination was associated with a 100% survival rate. Similar clinical improvements have been reported by Freedberg et al [119] among hospitalized COVID-19 patients. Despite clinical evidence suggesting that famotidine may mitigate COVID-19, its mechanism of action remains a matter of debate. A recent study by Malone et al [120] suggests that the drug’s therapeutic action in COVID-19 involves on-target histamine receptor-H2 activity, which has face validity since the development of clinical symptoms involves dysfunctional mast cell activation and histamine release.

### Fenofibrate

Researchers from the Hebrew University of Jerusalem, Israel, and Icahn School of Medicine at Mount Sinai (United States) studied the metabolic changes induced by SARS-CoV-2 infection in bronchial epithelial cells using lung biopsy samples from patients with COVID-19. The researchers reported a significant metabolic response in SARS-CoV-2–infected lungs in addition to changes in lipid metabolism and the induction of inositol-requiring enzyme-1 and RNA-activated protein kinase pathways of endoplasmic stress. The study showed that fenofibrate reversed the metabolic changes induced by SARS-CoV-2, blocking viral production and suppressing the pathogenesis of COVID-19 in lung tissue [121].

### Sildenafil

A recent systematic review carried out by researchers from the University of Rome, Italy, consolidated evidence of the involvement of the NO-cGMP-PDE5 axis in the pathophysiology of COVID-19, presenting ongoing clinical trials aimed at modulating this axis, including the DEDALO (silDEnafil administration in DiAbetic and dysmetaboLic patients with COVID-19) trial [122]. Reviewed data indicate that PDE5 inhibitors could be effective in managing patients with COVID-19 by counteracting the Ang-II–mediated downregulation of the AT-1 receptor, exhibit action on monocyte switching, reducing proinflammatory cytokines and interstitial infiltration; and inhibit the transition of endothelial and smooth muscle cells to mesenchymal cells in the pulmonary artery, preventing clotting and thrombotic complications. With sildenafil’s low cost, well-established safety, wide availability, and efficacy arising from observational studies and clinical trials (including the new “Sildenafil in COVID-19” trial; ClinicalTrials.gov ID NCT04489446), it, and other PDE5 inhibitors, could potentially become key COVID-19 treatment options [122,123].

## Abbreviations

ARDS: acute respiratory distress syndrome
bid: twice daily
cAMP: cyclic adenosine monophosphate
cGMP: cyclic guanine monophosphate
CKD: chronic kidney disease
CRP: C-reactive protein
CTAD: citrate-theophylline-adenosine-dipyridamole
DEDALO: silDEnafil administration in DiAbetic and dysmetaboLic patients with COVID-19
FDA: Food and Drug Administration
GERD: gastroesophageal reflux disease
H_2_RA: histamine type-2 receptor antagonist
HSV: herpes simplex virus
NO: nitric oxide
PAH: pulmonary arterial hypertension
PDE: phosphodiesterase-5
qid: four times daily
